# Annotations

**Published:** 1904-01-02

**Authors:** 


### Annotations.

### Charity and Wisdom.

The newspapers are advertising as a "notable act of seasonable generosity" the gift of £1,500 made by a Surrey gentleman for Christmas gifts to the indoor and outdoor paupers of the county where he lives. One willingly gives him all credit for his kindness of intention, but when we go on to read that each pauper will in all probability receive two shillings as his share, we wonder if in this case wisdom and kindness have gone hand in hand. A dole of two shillings will hardly lift the best-intentioned pauper out of pauperdom, and will not add a very great deal to his seasonable festivity. Yet £1,500 is a large sum. If invested it would, at the most moderate calculation, produce an income of £45 a year, and this in wise hands might lift a few families out of temporary distress and put them again among the ranks of the self-supporting. To keep even one family a year out of the ranks of pauperdom—and wisely expended the money would do more than this—would be a far greater boon than to add a little exhilaration to those who have fallen into that class. Very rarely are doles of any real good. Busy people are almost compelled to do their almsgiving by proxy, but at least they can think out on what lines their donations will be spent, remembering always that it is as possible to do harm as good by even a "seasonable gift." Hard though the saying may seem, charity should be an investment—something which it is expected will give a good return in, if possible, lifting the recipient above the need of outside aid in future. It may be necessary to give permanent help to the aged and infirm, according to their needs, but this also means the concentration of a large sum upon a few rather than lavishing it in a temporary help to many.

### The Medico-Legal Society.

This is the latest addition to the numerous societies concerned with the scientific and practica interests of medicine. It has successfully achieved its first session, and now enters on its second year's programme. This contains some very important and interesting items, and argues well for the success of the purpose which the society has in view. It includes discussions on questions of general jurisprudence such as juries and expert evidence, executive questions as employers' liability and the Public Control Department of the L.C.C., as well as papers and demonstrations on forensic medicine in the more strict sense of the term. It is proposed to develop the practical usefulness of the society by inviting the exhibition of specimens and the submission of urgent points of importance at each meeting before the authorised programme is entered upon. In connection with this "clinical" development of the society's work we notice that on February 9th Mr. J. H. Targett is to show the specimens from a case of homicide by the introduction of foreign bodies, and Professor Bostock Hill is to relate a case of attempted abortion by drugs. Medico-legal difficulties are apt to make a very urgent and strongly personal appeal to those who have to deal with them and it is certainly to the public advantage that the two professions most intimately concerned should have a common meeting ground. The society thus appeals to a very large constituency and deals with matters which have both a scientific and keenly human interest. There can be little doubt that it will soon command a large success. Its ambition ought not to be confined to a mere programme of meetings, valuable and important as these may be. There are numerous questions, such for example as the registration of deaths, which are of enormous social importance and on which a medico-legal society ought to be able to speak with peculiar competence. The transactions of such a society ought to be an authoritative chronicle of facts and principles illustrating the territory where medicine and jurisprudence meet, and we would suggest that the development of a museum should be included in the society's programme. The opportunity in this and allied directions is a large one, as medico-legal experts well know. The president for the current session is Sir William Collins, and the honorary secretary Mr. R. Henslowe Wellington of the Middle Temple.

### Moral Education of Children.

Much attention has recently been devoted to the study of moral defects in children, and a great deal of controversy has been aroused on the subject. That the discussion of the cause of children's faults will do good by making parents pay more attention than many do to the individualities of their little ones is certain, but on the other hand there is always a risk that the notion that their defects are the outcome of circumstances—pathological or other—over which the little offenders have no control, may tend to weaken the power of nursery discipline. To regard every naughty child as morally defective is a cruel kindness. A child is naughty sometimes from mere high spirits, sometimes from imagination, sometimes from the inevitable narrowness of childhood's experience and outlook. Time and the give-and-take of life will cure these faults, but for the sake of peace in the home it may be advisable to hasten the teachings of experience by means of some of the traditional nursery punishments. These may not all have a direct moral bearing, but if cause and effect be duly considered, they will prove to the culprit that a certain course of conduct "does not pay," and this is as much as many children, and some adults, can understand. As the child grows older it will be possible to explain to him the moral reasons on which we base our approval or disapproval of a certain course of action, and inculcate justice, kindness, and self-control. Unless these things are taught, the training of the child, whether the means employed be old-time harshness or modern tenderness, is a failure. Even in dealing with those who are admittedly morally defective it is well to dwell on these points, to assume for them a virtue which they only imperfectly possess. Nothing could be worse for the average child, who is not defective, but generally, within the limits of his experience, a very shrewd person, than to get an inkling that his parents, nurses, or teachers do not consider him responsible for his acts of destruction and disobedience, for his fibs and petty thefts.

